# LncRNA SNHG16 as a potential biomarker and therapeutic target in human cancers

**DOI:** 10.1186/s40364-020-00221-4

**Published:** 2020-09-10

**Authors:** Yuhang Xiao, Ta Xiao, Wei Ou, Zhining Wu, Jie Wu, Jinming Tang, Bo Tian, Yong Zhou, Min Su, Wenxiang Wang

**Affiliations:** 1 Thoracic Surgery Department 2, Hunan Cancer Hospital and The Affiliated Cancer Hospital of Xiangya School of Medicine, Central South University, Changsha, Hunan 410013 PR China; 2 Department of Pharmacy, Xiangya Hospital of Xiangya School of Medicine, Central South University, Changsha, Hunan 410001 PR China; 3 Institute of Dermatology, Chinese Academy of Medical Sciences & Peking Union Medical College, Nanjing, Jiangsu 210042 China; 4 Department of Pharmacy, The First People’s Hospital of Yue Yang, Yue Yang, PR China; 5 Hunan Key Laboratory of Translational Radiation Oncology, Hunan Cancer Hospital and The Affiliated Cancer Hospital of Xiangya School of Medicine, Central South University, Changsha, China

**Keywords:** SNHG16, Long non-coding RNA, Human cancer, Mechanism, Biomarker

## Abstract

Long non-coding RNAs (lncRNAs) represent an important class of RNAs comprising more than 200 nucleotides, which are produced by RNA polymerase II. Although lacking an open reading framework and protein-encoding activity, lncRNAs can mediate endogenous gene expression by serving as chromatin remodeler, transcriptional or post-transcriptional modulator, and splicing regulator during gene modification. In recent years, increasing evidence shows the significance of lncRNAs in many malignancies, with vital roles in tumorigenesis and cancer progression. Moreover, lncRNAs were also considered potential diagnostic and prognostic markers in cancer. The lncRNA small nuclear RNA host gene 16 (SNHG16), found on chromosome 17q25.1, represents a novel tumor-associated lncRNA. SNHG16 was recently found to exhibit dysregulated expression in a variety of malignancies. There are growing evidence of SNHG16’s involvement in characteristics of cancer, including proliferation, apoptosis, together with its involvement in chemoresistance. In addition, SNHG16 has been described as a promising diagnostic and prognostic biomarker in cancer patients. The current review briefly summarizes recently reported findings about SNHG16 and discuss its expression, roles, mechanisms, and diagnostic and prognostic values in human cancers.

## Introduction

Cancer remains an important cause of death worldwide, with increasing incidence and mortality [1, 2]. There are approximately 18.1 million new cancer cases and 9.6 million cancer-related deaths worldwide in 2018 [3]. In recent years, although the understanding of molecular mechanisms underlying cancer has increased substantially [4–7], and treatments for cancer have greatly developed [8, 9], the recurrence and mortality rate of cancer patients are still pessimistic [1]. Therefore, it is imperative to find novel and effective biomarkers and therapeutic targets.

Several recent reports have shown that non-coding RNAs (ncRNAs) contribute to tumorigenesis [10]. NcRNAs are generally divided into two categories by length, including small ncRNAs with length below 200 nucleotides (nts) and long ncRNAs (lncRNAs), which have more than 200 nts [11, 12]. MicroRNAs (miRNAs), a kind of small ncRNAs with 20 to 25 nucleotides, have been shown to negatively regulate the expression of particular key genes and participate in various aspects of cell biology [13, 14]. LncRNAs have attracted increasing attention in recent years. Although lncRNAs represent transcripts without protein-coding potential, they could control gene expression at multiple levels with epigenetic (e.g., DNA methylation, histone modifications, and chromatin remodeling), transcriptional (e.g., recruitment of RNA polymerase II, transcription factors and cofactors), and post-transcriptional (e.g., sponging of miRNAs, alternative splicing, regulating mRNA stability, regulating translation, interacting with proteins) regulations [15–17]. Increasing evidence suggests that many lncRNAs show abnormal expression levels in various tumors [18]. LncRNA dysregulation is generally involved in pertinent tumor cellular events, including growth, programmed cell death, metastasis, and stemness [19].

The lncRNA small nuclear RNA host gene 16 (SNHG16) (also named as non-coding RNA expressed in aggressive neuroblastoma [ncRAN]), which contains 2435 nts, is located on chromosome 17q25.1 [20]. There are two splicing variants of SNHG16, including the long form Nbla10727 (with length of 2186 nts) and the short form Nbla12061 (with length of 2087 nts) [21]. SNHG16 was firstly described as a potent oncogenic factor causing poor patient outcome in neuroblastoma [20]. The latter study also showed that elevated SNHG16 amounts contribute to the amplification of the v-myc myelocytomatosis viral related oncogene, neuroblastoma derived (avian) (MYCN) (2p24) locus in this malignancy [20]. Subsequent studies showed that SNHG16 was deregulated and function in several cancers. This review summarizes recently reported findings about SNHG16’s roles in malignancies, including those describing the abnormal expression, molecular functions, and regulatory network of SNHG16, in addition to associated clinical features.

## Expression of SNHG16 in cancer

SNHG16 expression is typically increased in a variety cancers, including acute lymphoblastic leukemia, bladder cancer, breast carcinoma, cervical and ovarian cancers, B-cell lymphoma, endometrial carcinoma, esophageal cancer, gastric cancer, glioma, neuroblastoma, lung carcinoma, mouth cancer, osteosarcoma, pancreatic cancer, papillary thyroid cancer, and retinoblastoma (Table 1). Cell lines established from these malignancies also show trends of SNHG16 upregulation. However, in colorectal cancer and hepatocellular carcinoma, there are contrary reports of SNHG16 expression. In hepatocellular carcinoma, the first report by Xu and colleagues [44] demonstrated that SNHG16 amounts are reduced in liver cancer tissue samples and cells. These authors speculated that the site and microenvironment of a given malignancy might provide explanations regarding differences in SNHG16 expression compared with other human cancers. However, after that, at least 5 studies have reported that SNHG16 is upregulated in liver cancer tissue specimens and cells [45, 46]. In colorectal cancer, two reports by Christensen and colleagues [31] and Li and collaborators [30] revealed SNHG16 upregulation in colorectal cancer tissue specimens and cells. On the contrary, another study by Qi et al. [32] reported SNHG16 downregulation in colorectal cancer. Christensen et al. [31] analyzed the above discrepancies in colorectal cancer, and suggested that the methods applied and assessed isoforms are improbable explanations, while RNA quality, tumor cell ratio in tissues, and variations in the used cells could explain these inconsistencies. In the report by Christensen et al., high-quality RNA was tested (median RIN score > 8.9), tumor cells comprised averagely 75% of colorectal cancer tissues, and the HCT116 cell line used was authenticated; these data were not reported by Qi and collaborators.

**Table 1:** Expression of SNHG16 in various cancers

Cancer type	Expression in tissue	Sample size	Expression in cancer cells	Cancer cell lines	Relative normal cell lines	Ref.
Acute lymphoblastic leukemia	up	37	up	MOLT3, MOLT4, SUP-B15, CCRF-CEM, RS4;11, TALL104, CEM/C1, CEM/C2, Loucy	BMMC	[22]
Bladder cancer	up	275	up	5637, J82, RT4, T24	SV-HUC-1	[23]
	up	26	up	T24, RT4, 253 J-Bv	SV-HUC-1	[24]
	up	46	up	T-24, BIU87, 5637	SV-HUC-1	[25]
	up	40	up	5637, T24, J82	RT4	[26]
Breast cancer	up	30	–	MDA-MB-231, MCF-7, MDA-MB-468, HEK293T	–	[27]
Cervical cancer	up	38	up	SiHa, CaSki, HeLa, C33A	H8	[28]
	up	66	up	SiHa, CaSki, HeLa, C33A, ME180	HcerEpic	[29]
Colorectal cancer	up	56	up	SW480, LoVo, CaCO-2 and HCT116	CCC-HIE-2	[30]
	up	314	up	HCT116, SW480	–	[31]
	down	81	down	LoVo, Caco-2, DLD1, SW620, SW480, HCT8, HCT116	CCC-HIE-2	[32]
Diffuse large B-cell lymphoma	up	48	up	OCI-LY7, OCI-LY3	B lymphocytes	[33]
Endometrial carcinoma	up	–	up	HEC-1B, HEC-1A, RL95–2, AN3CA	MEC	[34]
Esophageal squamous cell carcinoma	up	68	up	eca109, EC9706, TE1, kyse-30, kyse-70	HEEC	[35]
	up	128	up	TE-13, TE-1, EC-1, Eca-109	HEEC	[36]
Gastric cancer	up	32	up	BGC823, MGC803, MKN45, SGC7901	GES-1	[37]
	up	122	up	AGS, BGC-823, MGC-803, SGC-7901, MKN451	GES-1	[38]
	up	20	up	HGC-27 and AGS	–	[39]
	up	54	–	–	–	[40]
Glioma	up	48	up	U251, H4, SW1783, LN229	NHAs	[41]
	up	40	up	U251, H4, SW1783, LN229	NIHAS	[42]
	up	40	up	U251, LN229	NHA	[43]
Hepatocellular carcinoma	down	43	down	Hep3B, HuH7, SNU398, SNU423, SNU429, Hep3G2, SK-HEP-1, PLC/PRF/5	THLE2, THLE3	[44]
	up	10	up	SK-Hep-1, Huh7, Hep3B, HepG2	HL-7702	[45]
	up	34	up	Huh7, HepG2, SMMC7721, SK-Hep1, Hep 3B	LO2	[46]
	up	47	up	MHCC97H, HuH7, SMMC7721, Hep3B, HepG2	LO2	[47]
	up	40	up	HepG2, SMMC7721, Hep3B, Bel7402, Huh7	LO2	[48]
	up	30	up	Huh7, HepG2, SMMC7721, QGY-7703	HL-7702	[49]
	up	50	up	Hep-3B, Huh7, Sk-hep-1, SMMC-7721, PLC	HL-77O2	[50]
Neuroblastoma	up	48	up	SK-N-SH, IMR-32, SK-N-AS, SK-N-DZ	HUVEC	[51]
	up	40	–	–	–	[52]
Non small cell lung cancer	up	66	up	A549, NCI-H292, NCI-H460, NCI-H1703	16HBE	[53]
	up	30	up	A549, NCI-H292, NCI-H460, NCI-H1703	16HBE	[54]
Oral squamouscell carcinoma	up	29	up	SCC-25, CAL-27, Tca8113, TSCCA	NHOK	[55]
Osteosarcoma	up	25	up	U2OS, SaOS2	hFOB1.19	[56]
	up	96	up	U2OS, Saos-2, HOS, MG-63	hFOB 1.19	[57]
	up	20	up	MG-63, U2OS, SAOS2, HOS	OB3	[58]
	up	65	–	U2OS, MG-63	–	[59]
Ovarian cancer	up	103	up	SKOV-3, ES2, HO8910, OMC685	IOSE-29	[60]
Pancreatic cancer	up	46	up	BxPC-3, SW1990, PANC-1, AsPC-1	HPDE6-C7	[61]
Papillary thyroid cancer	up	48	up	IHH-4, TPC-1, HTH83	Nthy-ori 3–1	[62]
Retinoblastoma	up	30	up	WERI Rb1, SO-RB-50, Y79	ARPE-19	[63]

## Regulation of SNHG16 expression in cancer

Several transcription factors can regulate SNHG16 transcription in a positive manner, such as c-Myc, signal Transducer and Activator of Transcription 3 (STAT3), and transcription factor AP-2 alpha (TFAP2A). Li and co-workers [55] showed that SNHG16 is regulated by c-Myc, a transcription factor that exerts its function via recruitment of histone acetyltransferase and induction of RNA polymerase II clearance [64]. Meanwhile, Christensen and colleagues [31] demonstrated that SNHG16 is positively associated with the levels of transcription factors controlled by Wnt in colorectal cancer. β-catenin knockdown results in decreased SNHG16 and c-Myc amounts [31]. In addition, SNHG16 overexpression abrogated the cell function changes caused by c-Myc knockdown. In another study, Christensen et al. [31] found that SNHG16 was positively associated with the expression of the Wnt-regulated transcription factors in colorectal cancer. β-catenin knockdown resulted in decreased expression of SNHG16 and c-Myc [31]. In addition, c-Myc knockdown resulted in SNHG16 downregulation, while its upregulation markedly increased SNHG16 expression [31]. These results strongly indicate that Wnt signaling-controlled transcription factors, including c-Myc, could regulate SNHG16 expression. In addition to c-Myc, STAT3 could also influence SNHG16 expression [31]. Small interfering RNA (si-RNA) knockdown of STAT3 resulted in decreased SNHG16 expression in colorectal cancer cells. Moreover, transcription factor TFAP2A could directly bind with the SNHG16 promotor region and activate its transcription of SNHG16 [34].

## Functional activities of SNHG16 in cancer

Evidence suggests that several events contribute to malignant features of cells such as sustained growth, resistance to death, induced invasion and metastasis, and increased chemotherapeutic resistance. The hallmarks of cancer are due to mutations producing oncogenes and tumor suppressors. In recent years, SNHG16 has been highlighted with its important functions in mediating these oncogenes and tumor suppressors and generally regulating these cancer characteristics (Table 2).

**Table 2:** In vitro functional characterization of SNHG16 in cancer

Cancer type	Effect on viability/proliferation	Effect on apoptosis	Effect on invasion/metastasis	Effect on chemoresistance	Ref.
Acute Lymphoblastic Leukemia	promote	–	promote	–	[22]
Bladder cancer	promote	inhibit	promote	–	[24–26]
Breast cancer	–	–	promote	–	[27]
Cervical cancer	promote	–	promote	–	[28, 29]
Colorectal cancer	promote	inhibit	promote	–	[30, 31]
Diffuse large B-cell lymphoma	promote	inhibit	–	–	[33]
Endometrial carcinoma	promote		–	–	[34]
Esophageal squamous cell carcinoma	promote	inhibit	promote	–	[35, 36]
Gastric Cancer	promote	inhibit	promote	–	[35, 37, 39, 40]
Glioma	promote	inhibit	promote	–	[41–43]
Hepatocellular carcinoma	inhibit	–	–	inhibit	[44]
	promote	inhibit	promote	promote	[45–50]
Neuroblastoma	promote	inhibit	promote	–	[20, 51, 52]
Non-small cell lung cancer	promote	inhibit	promote	–	[54]
Oral squamous cell carcinoma	promote	inhibit	promote	–	[55]
Osteosarcoma	promote	inhibit	promote	–	[56–59]
Ovarian cancer	promote	–	promote	–	[60]
Pancreatic cancer	promote	–	promote	–	[61]
Papillary thyroid cancer	promote	inhibit	promote	–	[62]
Retinoblastoma	promote	inhibit	–	–	[63]

### SNHG16 in cell proliferation

Clearly SNHG16 could regulate the growth and proliferation of various cell lines, and thus regulate the growth of cancers in xenograft models (Table 3). SNHG16 expression is tightly associated with tumor size in several human cancer types. The pan-cyclin-dependent kinases (CDK) inhibitor p21 blocks the cell cycle at G0/G1. SNHG16 knockdown in bladder cancer cells could significantly induce cell cycle arrest at G1 by increasing p21 expression [25]. In agreement, cell cycle arrest at G1 was reported in glioma cells following SNHG16 silencing, with si-SNHG16 exerting its effects via p21 upregulation and cyclin D1 and cyclin B1 downregulation [42].

**Table 3:** In vivo functional characterization of SNHG16 in cancer

Cancer type	Cell line	Animal model	Role in tumor growth	Ref.
Acute Lymphoblastic Leukemia	MOLT3	BALB/c nude mice	promote	[22]
Cervical cancer	HeLa	BALB/c nude mice	promote	[29]
Colorectal cancer	LoVo	BALB/c nude mice	promote	[30]
Diffuse large B-cell lymphoma	OCI-LY7	NOD/SCID mice	promote	[33]
Esophageal squamous cell carcinoma	kyse-70	BALB/c nude mice	promote	[35]
Gastric cancer	MGC-803	BALB/c nude mice	promote	[38]
Hepatocellular carcinoma	HuH7	athymic nude mice	inhibit	[44]
	HuH7	BALB/c nude mice	promote	[47]
	HepG2	BALB/c nude mice	promote	[48]
	HepG2	athymic nude mice	promote	[49]
	Hep-3B	BALB/c nude mice	promote	[50]
Neuroblastoma	SK-N-SH	BALB/c nude mice	promote	[51]
Non small cell lung cancer	A549	BALB/c nude mice	promote	[53, 54]
Oral squamous cell carcinoma	TSCCA	BALB/c nude mice	promote	[55]
Pancreatic cancer	AsPC-1	BALB/c nude mice	promote	[61]
Retinoblastoma	Y79	BALB/c nude mice	promote	[63]

### SNHG16 in cell apoptosis

Apoptosis, autophagy, and necrosis are three major pathways leading to cell death [16, 65]. Multiple reports have demonstrated that SNHG16 inhibits apoptosis in different cancers. These studies demonstrated SNHG16 knockdown induced cell apoptosis by flow cytometry detection. In bladder cancer cells, si-SNHG16 upregulates Bax and caspase-3, while downregulating Bcl-2 [23, 24]. Inhibition of SNHG16 could also induced more cleavage and activation of caspase-3 in glima [41] and oral squamous cell carcinoma cells [55]. In addition, in osteosarcoma [56], pediatric neuroblastoma [52], and colorectal cancer cells [31], downregulation of SNHG16 increased caspase 3/7 activity.

### SNHG16 in cancer metastasis

Metastasis represents a common cause of death in most cancers other than the primary tumor. SNHG16 affects cancer metastasis predominantly by controlling epithelial-to-mesenchymal transition (EMT). Knockdown of SNHG16 inhibits EMT in bladder cancer [23], colon cancer [30], esophageal cancer [35], cervical cancer [28], oral squamous cell carcinoma [55], gastric cancer [39], and hepatocellular carcinoma [47], and regulates EMT-associated molecules (upregulates E-cadherin, and downregulates N-cadherin and vimentin).

### SNHG16 in chemotherapeutic resistance

Currently, the chemotherapeutic treatment of many cancers is mainly hampered by drug resistance. SNHG16 contributes to chemoresistance in hepatocellular carcinoma. It was shown that siRNA knockdown of SNHG16 reverses sorafenib resistance in Hep3B and HepG2 cell lines [45], and sh-SNHG16 knockdown increases HuH7 and Hep3B cell sensitivities to cisplatin [47], but the underlying mechanisms were not clarified by these two studies. However, another study reported that upregulation of SNHG16 inhibits 5-fluorouracil (5-FU) chemoresistance in Hep3B and HuH7 cells [44], which contrasts the two reports mentioned above. Moreover, tissue and cell SNHG16 levels in the latter report [14] were also different from those reported by several other studies; thus, these findings need further validation.

## Mechanisms of SNHG16 action in cancer

**Fig. 1:**
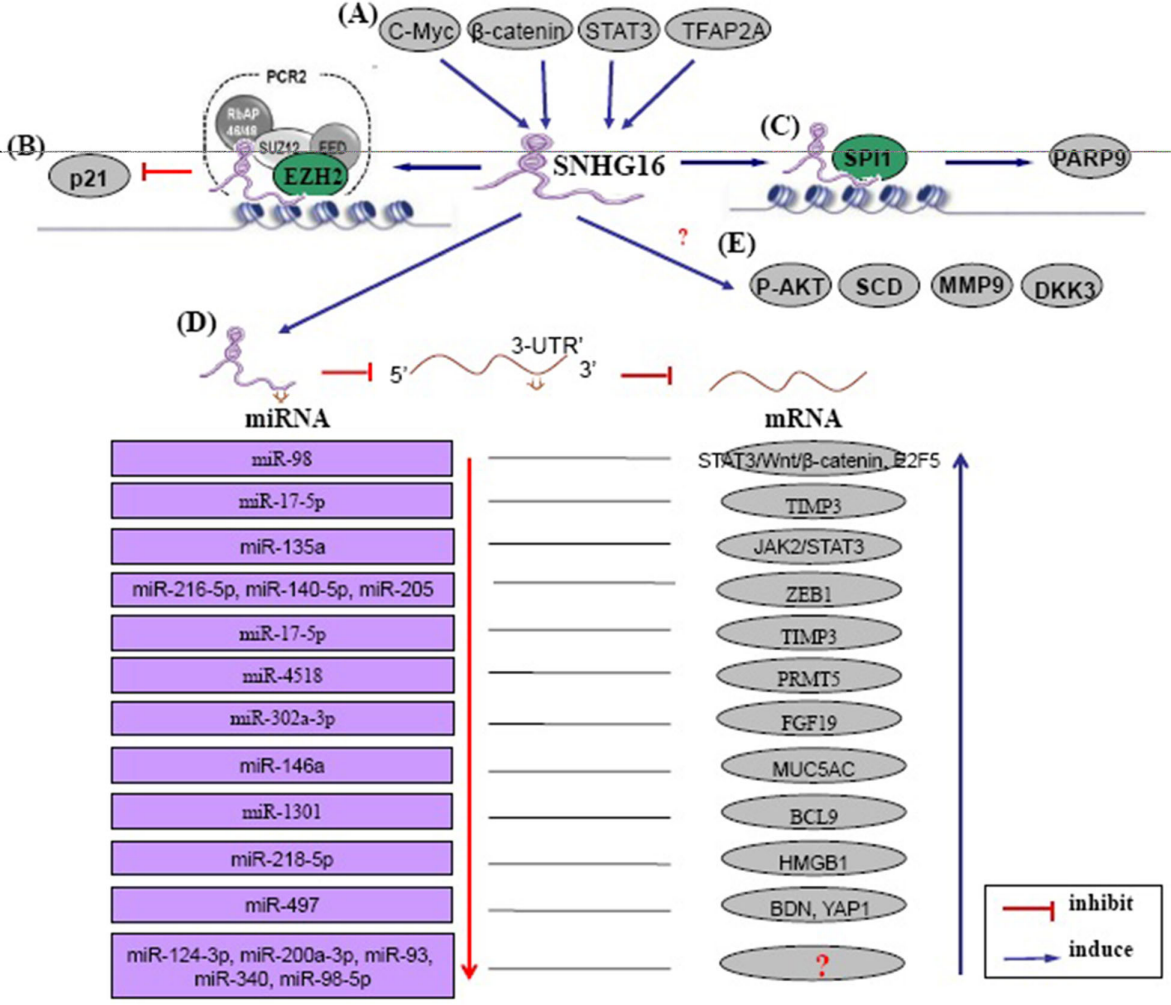
Upstream regulatory and downstream molecular mechanisms underlying SNHG16 in human cancers. **a** SNHG16 is positively regulated by transcription factors, such as c-Myc, β-catenin, STAT3, and TFAP2A; **b** SNHG16 binds to EZH2 and recruits EZH2 to p21 promoter, subsequently inhibits p21 expression; **c** SNHG16 binds to SPI1/PU.1 and recruits SPI1/PU.1 to PARP9 pomoter, subsequently upregulates PARP9 expression; **d** SNHG16 functions as a ceRNA to regulate multi miRNAs and target genes; **e** SNHG16 regulates expression of SCD, p-AKT and MMP9, DKK3, and Wnt/β-catenin, with uncovered mechanisms

Emerging evidence reveals that the modulatory effects of lncRNAs strongly depend upon their location in cells [66]. LncRNAs located in the cytoplasm may regulate target genes in a post-transcriptional pathway by serving as partners with miRNA [67]. It is widely admitted that SNHG16 is primarily expressed in the cytoplasm of cells [31, 34, 35, 37, 45, 49], and thus acts as a competing endogenous RNA (ceRNA) to affect the expression of key targets. SNHG16 was also reported to be obviously expressed in nucleus [25], and possibly exert its function at the transcriptional level (Fig. 1).

### SNHG16 serves as ceRNA

CeRNA is defined as ncRNAs interact with miRNAs through miRNA response elements (MRE) to inactivate and release the repression of miRNAs to its the target genes. A number of studies demonstrated that SNHG16 functions as a ceRNA to regulate multi miRNAs, including miR-124-3p [22], miR-98 [24, 57], miR-216-5p [28], miR-200a-3p [30], miR-140-5p [35], miR-135a [37], and so on (Table 4). In these studies, luciferase reporter assays and RNA immunoprecipitation (RIP) and/or RNA pull-down assays were carried out to reveal a miRNA-binding site in the SNHG16 sequence, and functional assays suggested that the miRNA and associated target genes control SNHG16’s effects.

**Table 4:** CeRNA function of SNHG16 in cancer

SNHG16 Target miRNA	Validated method	miRNA Target gene	Cancer type	Ref.
let-7b-5p	luciferase reporter assay, RIP	CDC25B/CDK1	hepatocellular carcinoma	[47]
miR-124-3p	luciferase reporter assay	–	acute Lymphoblastic Leukemia	[22]
miR-128-3p	luciferase reporter assay, RIP	HOXA7	neuroblastoma	[51]
miR-1301	luciferase reporter assay, RNA pull down	BCL9	osteosarcoma	[59]
miR-135a	luciferase reporter assay, RIP	JAK2/STAT3	gastric Cancer	[37]
miR-140-5p	luciferase reporter assay, RIP, RNA pull down	ZEB1	esophageal squamous cell carcinoma	[35]
	luciferase reporter assay, RIP	–	retinoblastoma	[63]
miR-146a	luciferase reporter assay, RIP	MUC5AC	non small cell lung cancer	[53]
miR-17-5p	luciferase reporter assay	TIMP3	bladder cancer	[23]
	luciferase reporter assay, RIP	p62	hepatocellular carcinoma	[49]
miR-186	luciferase reporter assay	–	hepatocellular Carcinoma	[50]
miR-195	luciferase reporter assay, RIP	–	hepatocellular carcinoma	[48]
miR-200a-3p	luciferase reporter assay	–	colorectal cancer	[30]
miR-205		ZEB1	osteosarcoma	[58]
miR-216-5p	luciferase reporter assay, RIP, RNA pull down	ZEB1	cervical cancer	[28]
miR-218-5p	luciferase reporter assay, RNA pull-down	HMGB1	pancreatic cancer	[61]
miR-302a-3p	luciferase reporter assay, RNA pull-down	FGF19	hepatocellular carcinoma	[46]
miR-340	luciferase reporter assay	–	osteosarcoma	[56]
miR-373	luciferase reporter assay	EGFR	glioma	[43]
miR-4518	luciferase reporter assay	PRMT5	glioma	[41]
miR-490-3p	luciferase reporter assay	HK2	endometrial carcinoma	[34]
miR-497	luciferase reporter assay	BDN, YAP1	papillary thyroid cancer	[62]
miR-497-5p	luciferase reporter assay, RIP, RNA pull down	PIM1	diffuse large B-cell lymphoma	[33]
miR-520a-3p	luciferase reporter assay	EphA2	non small cell lung cancer	[54]
miR-628	luciferase reporter assay	–	gastric cancer	[40]
miR-93	luciferase reporter assay	–	hepatocellular carcinoma	[44]
miR-98	luciferase reporter assay	STAT3	bladder cancer	[24]
	luciferase reporter assay, RIP	E2F5	breast cancer	[27]
miR-98-5p	luciferase reporter assay, RIP	–	osteosarcoma	[57]

### SNHG16 interacts with proteins

LncRNAs can interact with proteins to modulate its function. SNHG16 has been reported to directly bind to Enhancer of Zeste Homolog 2 (EZH2) and recruits it to the promoter regions of p21, which subsequently induces histone modification [25]. Another recent study demonstrated that SNHG16 upregulates poly(ADP-ribose) polymerases 9 (PARP9) expression through binding and recruiting transcription factor SPI1/PU.1 to PARP9 pomoter [29].

### SNHG16 regulates various genes

In addition, there are some molecules reported to be mediated by SNHG16, including Stearoyl-CoA Desaturase (SCD) [31], p-AKT and matrix metallopeptidase 9 (MMP9) [60], Dickkopf-related protein 3 (DKK3) [39], and Wnt/β-catenin [36], but the underlying regulate mechanisms are not well uncovered. Furthermore, whether SNHG16 has other regulatory functions still needs further investigation.

## SNHG16 as a biomarker in cancer

### SNHG16 serves as a diagnostic biomarker in cancer

LncRNA detection in body fluids, including serum and plasma specimens, provides a novel avenue for early noninvasive cancer diagnosis. SNHG16 was shown to be relatively stable under freeze-thaw cycles or long-term storage, indicating an advantage for SNHG16 as a potentially effective diagnostic biomarker in malignancies [68]. SNHG16’s diagnostic value was investigated in bladder cancer, with elevated plasma, serum, and serum exosome amounts detected in bladder cancer cases compared with healthy controls. Indeed, SNHG16 was shown to be markedly elevated in plasma specimens from 26 bladder cancer cases vs. 15 control patients [24], as well as in serum samples from 120 bladder cancer cases vs. 52 healthy subjects [68]. However, the diagnostic value of SNHG16 was not evaluated in the two above studies, as areas under the receiver operating characteristic (ROC) curves (AUC values) were not shown. In another study, Zhang and collaborators [69] reported increased SNHG16 amounts in the serum exosomes of 100 breast cancer cases in comparison with 100 healthy controls. The AUC was 0.681, which indicates that the accuracy of breast cancer classification based on SNHG16 is low. To date, there is no evidence indicating that SNHG16 alone might constitute an ideal diagnostic biomarker in cancer.

The diagnostic value of SNHG16 was further investigated in combination with other lncRNAs in bladder cancer. Duan and colleagues [68] obtained an AUC for a three-lncRNA panel (SNHG16, MEG3, and MALAT1) of 0.865 in the training set comprising 240 serum samples (52 healthy individuals, and 68 non-cancer and 120 bladder cancer cases) and 0.828 in the validation set composed of 200 serum samples (48 healthy individuals, and 52 non-cancer and 100 bladder cancer cases). This three-lncRNA combination showed AUC values for Ta, T1, and T2-T4 of 0.778, 0.805, and 0.880, respectively, upon serum detection. In another study, Zhang et al. [69] identified a lncRNA panel in serum exosomes for diagnosing and predicting recurrence in bladder cancer using a training set of 100 serum exosome samples (50 healthy controls and 50 bladder cancer patients) and a validation cohort of 320 serum exosome specimens (160 healthy controls and 160 bladder cancer patients). They reported, in the training cohort, an AUC for a three-lncRNA combination (SNHG16, PCAT-1 and UBC1) of 0.857; the accuracy of this lncRNA panel to differentiate between healthy individuals and bladder cancer patients was 0.815 (sensitivity and specificity of 0.85 and 0.78, respectively). The corresponding AUC values for the lncRNA combination in Ta, T1, and T2-T4 cases were 0.760, 0.827, and 0.878, respectively. In the validation cohort, an AUC for the three-lncRNA combination of 0.826 was obtained, and sensitivity and specificity were 0.80 and 0.75, respectively. Moreover, the three-lncRNA combination had an AUC significantly elevated in comparison with urine cytology (0.574), a biomarker with commendable sensitivity in detecting high- but not low-grade bladder cancers, indicating the potential of the above lncRNA panel as an effective biomarker for bladder cancer diagnosis. Collectively, the above findings suggested that the SNHG16-combined-lncRNA panels could differentiate bladder cancer patients from healthy individuals with great accuracy, which warrants further multicenter trials with larger clinical samples for confirmation. However, SNHG16 is found in multiple cancer types and less likely to help distinguish the specific origins of tumors. Thus, further studies should be performed to analyze the diagnostic value of SNHG16 in body fluids in combination with other specific biomarkers in different cancers.

### SNHG16 serves as a prognostic marker in cancer

It was shown that abnormal SNHG16 expression is closely related to cancer prognosis (Table 5). High SNHG16 amounts were shown to be significantly related to poor overall survival bladder cancer, cervical cancer, endometrial carcinoma, esophageal cancer, stomach cancer, glioma, liver cancer, neuroblastoma, lung cancer, osteosarcoma, and ovarian and pancreatic cancers. SNHG16 expression independently predicts prognosis in these cancers. Additionally, SNHG16 upregulation was also shown to be related to poor progression free survival (PFS) in glioma, liver cancer, and lung cancer. However, in colon cancer, it was reported that SNHG16 downregulation is related to shorter overall survival (OS) of patients. Apart from survival data, other clinical features related to SNHG16 expression have been reported. In bladder cancer, high tumor SNHG16 levels are significantly related to tumor stage, histological grade, and lymph node metastasis. In cervical cancer, SNHG16 overexpression is related to tumor size and differentiation, as well as lymph node metastasis and FIGO stage. In esophageal cancer, cases expressing high SNHG16 amounts show elevated tumor stage, clinical stage, and lymph node metastasis. In ovarian cancer, high levels of SNHG16 are linked to clinical stage and distant metastasis. In pancreatic cancer, SNHG16 upregulation is associated with TNM stage, tumor differentiation, and distant metastasis. High SNHG16 levels are also associated with tumor size and TNM stage in glioma and hepatocellular carcinoma. Jointly, the above findings indicate that SNHG16 might represent an independent parameter for predicting prognosis in various cancers, although further large trials are still required for confirmation.

**Table 5:** Involvement of SNHG16 in cancer prognosis

Cancer type	Prognostic indicator	Associated clinical features	Ref.
Bladder cancer	OS	tumor stage, histological grade, lymph node metastasis	[23, 25]
Cervical cancer	OS	tumor size, tumor differentiation, lymph node metastasis, FIGO stage	[28, 29]
Colorectal Cancer	OS	–	[32]
Endometrial carcinoma	OS, recurrence free survival	–	[34]
Esophageal squamous cell carcinoma	OS	tumor stage, clinical stage, lymph nodes metastasis	[36]
Gastric Cancer	OS	tumor size, tumor differentiation, TNM stage, lymph node metastasis	[37, 38]
Glioma	OS, PFS	tumor size, WHO grade	[41]
Hepatocellular carcinoma	OS, PFS	tumor size, TNM stage	[45, 46, 49, 50]
Neuroblastoma	OS, event free survival	–	[20, 52]
Non small cell lung cancer	OS, DFS	tumor size, TNM stage, lymph node metastasis	[53]
Osteosarcoma	OS	–	[57, 59]
Ovarian cancer	OS	clinical stage, distance metastasis	[60]
Pancreatic cancer	OS	TNM stage, tumor differentiation, distant metastasis	[61]

## Conclusions

Numerous studies have confirmed that lncRNAs have critical functions in human cancer formation and progression. SNHG16, also named ncRAN, was initially reported as a potent oncogene in neuroblastoma. Since then, SNHG16 upregulation has been demonstrated in major human cancers. However, published SNHG16 expression patterns vary, and studies assessing the role of SNHG16 have reported conflicting results even in the same type of cancer (e.g., colorectal or liver cancer). SNHG16 contributes to the regulation of biological events in cells such as proliferation, apoptosis, malignancy potential, and chemoresistance. Thus, SNHG16 may represent a potential therapeutic candidate in a variety of cancer types. Mechanistically, post-transcriptional regulation of genes via ceRNAs appears to be an important mechanism underlying the effects of SNHG16. SNHG16 is mostly found in the cytosol, and sponges multiple miRNAs (more than 25 miRNAs reported so far) for target gene regulation. However, the overall mechanism underlying SNHG16 dysregulation in malignancies is not yet completely understood. In relation to clinical practice, SNHG16 dysregulation shows associations with clinical phenotypes as well as patient survival in many cancers, suggesting that SNHG16 might be a prognostic marker. SNHG16 might also represent a promising noninvasive diagnostic marker in cancer in combination with other specific biomarkers, because it is found in body fluids, including plasma, serum, and serum exosomes. Taken together, data from previous reports and the The Cancer Genome Atlas (TCGA) database indicate the upregulation of and an oncogenic role for SNHG16 in various cancers, although discrepant findings require clarification. In addition, further research is needed to facilitate the translation of basic science research evaluating SNHG16 into clinical benefits.

## Data Availability

Not applicable.

## Abbreviations

5-FU: 5-fluorouracil
AUC: Areas under the receiver operating characteristic curves
CDK: Cyclin-dependent kinases
ceRNA: Competing endogenous RNA
DKK3: Dickkopf-related protein 3
EMT: Epithelial-to-mesenchymal transition
EZH2: Enhancer of Zeste Homolog 2
MMP9: Matrix metallopeptidase 9
miRNAs: MicroRNAs
MRE: MiRNA response elements
MYCN: v-myc myelocytomatosis viral related oncogene, neuroblastoma derived (avian)
ncRAN: Non-coding RNA expressed in aggressive neuroblastoma
ncRNAs: Non-coding RNAs
OS: Overall survival
PARP 9: Poly(ADP-ribose) polymerases 9
PFS: Progression free survival
RIP: RNA immunoprecipitation
ROC: Receiver operating characteristic
SCD: Stearoyl-CoA Desaturase
siRNA: Small interfering RNA
SNHG16: Small nuclear RNA host gene 16
STAT3: Signal Transducer and Activator of Transcription
TCGA: The Cancer Genome Atlas
TFAP2A: Transcription factor AP-2 alpha
